# Dauerhaftes Sporttreiben im Sportverein und motorische Entwicklung: Ergebnisse der MoMo-Längsschnittstudie (2003–2017)

**DOI:** 10.1007/s43594-021-00054-5

**Published:** 2021-11-22

**Authors:** Anke Hanssen-Doose, Doris Oriwol, Claudia Niessner, Steffen Christian Eckehard Schmidt, Katja Klemm, Alexander Woll, Annette Worth

**Affiliations:** 1 Institut für Bewegungserziehung und Sport, Arbeitsbereich Bewegungsbildung, Diagnostik und Sport, Pädagogische Hochschule Karlsruhe, Bismarckstr. 10, 76133 Karlsruhe, Deutschland; 2 Institut für Sport und Sportwissenschaft, Karlsruher Institut für Technologie, Gebäude 40.40, Engler-Bunte-Ring 15, 76131 Karlsruhe, Deutschland

**Keywords:** Sportverein, Motorische Fähigkeiten, Fitness, Gesundheit, Sozialstatus, Sports club, Sports association, Motor performance, Fitness, Health, Social status

## Abstract

Bereits im Kindes- und Jugendalter gilt die motorische Leistungsfähigkeit als wichtiger Gesundheitsmarker. Auf Basis von Daten der Motorik Modul-Studie wird in diesem Artikel längsschnittlich über die Jahre 2003–2017 untersucht, inwieweit sich Sportvereinsmitglieder, die konstant im Sportverein aktiv waren, hinsichtlich ihrer motorischen Entwicklung von denjenigen unterscheiden, die nie im Sportverein aktiv waren. Es wurden Daten aus drei Messwellen untersucht: T1 (2003–2006), T2 (2009–2012) und T3 (2014–2017). Aus insgesamt *N* = 1092 Teilnehmenden, von denen über T1 bis T3 Daten zur Motorik vorlagen, wurden all diejenigen mit konstanter Mitgliedschaft und Nicht-Mitgliedschaft im Sportverein über drei Messwellen ausgewählt. Das sind 46 % der Gesamtstichprobe (*N* = 498). Von den *N* = 498 Teilnehmer*innen (Alter T1: 8,9 ± 3,8 Jahre, T2: 15,1 ± 3,9 Jahre, T3: 20,3 ± 4,0 Jahre) waren 15 % dauerhafte Sportvereinsmitglieder mit Wettkampfengagement, 53 % dauerhafte Sportvereinsmitglieder ohne Wettkampfengagement sowie 32 % dauerhaft Sportvereinsabstinente. Zur Ermittlung der motorischen Leistungsfähigkeit wurden konditionelle und koordinative Fähigkeiten anhand des MoMo-Testprofils erhoben (Kondition: Standweitsprung, Liegestütz, Fahrrad-Ausdauertest, Koordination: Seitliches Hin- und Herspringen, Einbeinstand, Balancieren rückwärts). Die Unterschiede in der Entwicklung wurden anhand von alters- und geschlechtsadjustierten Perzentilen mittels Varianzanalysen mit Messwiederholung berechnet, mit dem Sozialstatus als Kovariate. Innerhalb der Sportvereinsmitglieder waren Teilnehmende mit niedrigem Sozialstatus deutlich unterrepräsentiert. Insgesamt betrachtet, ist die Entwicklung der koordinativen und konditionellen Fähigkeiten bei Sportvereinsmitgliedern als signifikant besser zu beurteilen im Vergleich zu Sportvereinsabstinenten (Modell Koordination * Sportverein: df = 3,870 | F = 2,931 | *p* = 0,021 | ETA = 0,015 | f = 0,123; Modell Kondition * Sportverein: df = 4 | F = 3,794 | *p* = 0,005 | ETA = 0,048 | f = 0,225). Die Ergebnisse untermauern die Wichtigkeit der Sportvereine für die motorische Entwicklung von Kindern, Jugendlichen und jungen Erwachsenen in Deutschland.

## Abstract (en)

In childhood and adolescence the motor performance is seen as important marker of health. Within the population-based Motorik Modul Study, the motor development of continual sports club members has been compared to non sports club members longitudinally. Three measurement waves have been analysed: T1 (2003–2006), T2 (2009–2012) and T3 (2014–2017). Of the total sample *N* = 1092 participants with data in T1, T2 and T3, only those with continual (non-)participation have been chosen (46%, *N* = 498, age T1: 8.9 ± 3.8 years, T2: 15.1 ± 3.9 years, T3: 20.3 ± 4.0 years): 15% continual sports club members with participation in competitive sports, 53% continual sports club members without participation in competitive sports and 32% non sports club members. The motor performance was measured by means of the MoMo test profile (condition: standing long jump, push ups, ergometer test, coordination: jumping side to side, static stand, balancing backwards). The data were transferred into age- and sex-related percentile values. We then caluculated anovas with the socioeconomic status as covariate. Individuals with lower socioeconomic status were underrepresented in the groups with contunual sports clubs membership. The motor development—condition and coordination—was better in continual sports club members compared to non sports club members (modell coordination * sports club: df = 3.870 | F = 2.931 | *p* = 0.021 | ETA = 0.015 | f = 0.123; modell condition * sports club: df = 4 | F = 3.794 | *p* = 0.005 | ETA = 0.048 | f = 0.225). Sports clubs are an important setting for the motor development of children, adolescence and young adults in Germany.

## Einleitung

Bereits im Kindes- und Jugendalter gilt die motorische Leistungsfähigkeit als Gesundheitsmarker (Blasquez Shigaki et al. 2020; Hanssen-Doose et al. 2021; Ortega et al. 2008). Eine gute motorische Leistungsfähigkeit in jungen Jahren geht mit einem geringeren akuten und zukünftigen Erkrankungsrisiko einher (Mintjens et al. 2018) sowie einer Senkung des Risikos, vorzeitig zu sterben (Högström et al. 2016; Sato et al. 2009).

Die motorische Leistungsfähigkeit besteht nach dem fähigkeitsorientierten Ansatz aus den Dimensionen Kraft, Ausdauer, Schnelligkeit, Koordination und Beweglichkeit (Bös 1987). Mit der motorischen Entwicklung ist die Entwicklung dieser Dimensionen über die Zeit gemeint mit der Perspektive auf die gesamte Lebensspanne (Clark und Whithall 1989; Munzert 2010; Willimczik und Singer 2009). Motorische Entwicklung baut auf motorischen Üb‑, Lern-, und Trainingsprozessen auf und ist darüber hinaus stark von Wachstums- und Reifeprozessen abhängig (Munzert 2010), die sich insbesondere in Kindes- und Jugendalter vollziehen. Über die Lebensspanne hinweg können sich einzelne Dimensionen verschieden und sogar gegenläufig entwickeln und Veränderungen können sowohl positiv als Zunahme oder negativ als Abnahme der motorischen Leistungsfähigkeit in Erscheinung treten (Willimczik und Conzelmann 1999). Eine Vielzahl von Faktoren beeinflusst die motorische Entwicklung im Zeitverlauf positiv oder negativ. Ein niedriger Sozialstatus gilt als negativer Einflussfaktor der motorischen Entwicklung (Bös et al. 2009), während ein körperlich-sportlich aktiver Lebensstil als positiver Einflussfaktor beschrieben wird (Bös et al. 2009).

Sportvereine sind traditionell wichtige Lebenswelten, um gemeinsam sportlich aktiv zu sein (Breuer et al. 2015; Mutz 2020). Nach Emrich et al. (2001, S. 98) sind Sportvereine „soziale Gebilde zum Nutzen freiwilliger Mitglieder, deren weltanschauliche Bindung sich primär in der Auffassung vom Wert und Nutzens des gemeinsamen Sporttreibens manifestiert“.

**Figure 1:**
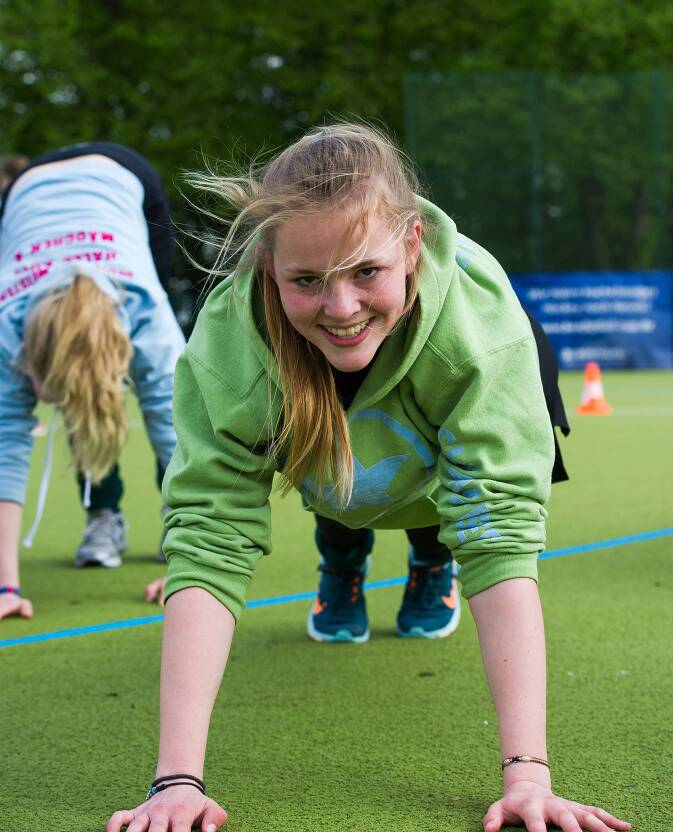

In Deutschland gibt es flächendeckend Sportvereine (Abb. 1) und diese bieten für die verschiedenen Altersgruppen umfassende Partizipationsmöglichkeiten. Das bedeutet nicht automatisch, dass jede*r in seiner*ihrer favorisierten Bewegungsform bzw. Sportart ein passendes Angebot findet. Insgesamt ist festzustellen, dass jüngere Zielgruppen das Angebot der Sportvereine als besonders attraktiv wahrnehmen: In Deutschland hat keine andere Organisation einen so hohen Anteil an Kindern und Jugendlichen in ihren Reihen (Breuer et al. 2015; Mutz 2020). Es gibt Hinweise darauf, dass Kinder und Jugendliche mit ihrer Freizeit zufriedener sind, wenn sie in mindestens einem Sportverein Mitglied sind (Schmidt 2020).

Sporttreibende finden in den Vereinen qualifizierte Übungsleiter*innen, ein breites Angebot an Sportarten und Bewegungsfeldern, aus dem sie interessensorientiert auswählen und Gleichgesinnte treffen können. Ob das Angebot aus der Perspektive benachteiligter Zielgruppen adäquat ist, also z. B. offen genug und ausreichend breit aufgestellt, hierzu ist die Datenlage dünn.

Sportvereine zeigen sich als anpassungsfähig, denn sie haben trotz Ganztagsschulentwicklung, G8-Gymnasium und zunehmender Konkurrenz bei der Freizeitgestaltung nach wie vor eine hohe gesellschaftliche Bedeutung (Breuer et al. 2015; Mutz 2020).

Dem organisierten Sport in der Altersgruppe der Jugendlichen wird mitunter jedoch eine gesundheitsabträgliche Wirkung zugeschrieben, weil es im Kontext des gemeinsamen Sporttreibens zu Alkoholkonsum kommen kann (Gerlach und Brettschneider 2013). Mit Initiativen und Programmen wie den Angeboten „Sport pro Gesundheit“, „Sport gegen Krebs“ oder „Deutsches Sportabzeichen“ liefern Sportvereine wiederum seit langem Impulse für die Förderung der Gesundheit ihrer Mitglieder. In kommunalen Netzwerken zur Gesundheitsförderung sind Vereine daher zunehmend interessante Partner (Wolff und Rütten 2013). Legt man den Setting-Ansatz der WHO zugrunde, so scheinen Sportvereine Public Health-relevant zu sein (Tiemann 2006). Aus der Sicht der Mitglieder von Sportvereinen sollten diese vordringlich innovativen Breiten- und Freizeitsport sowie Leistungs- und Wettkampfsport anbieten und den Solidaritätsgedanken umsetzen, d. h. einen Rahmen für das sportliche und gesellige Miteinander bereitstellen (Emrich et al. 2001). Obwohl die Gemeinwohlorientierung eine große Rolle spielt, wird eine Funktionalisierung der Sportvereine zur Kompensation gesundheitlicher Einschränkungen kritisch gesehen (Emrich et al. 2001).

Inwieweit Sportvereine ein Abbild der Gesellschaft sind, ist fraglich, denn Kinder, Jugendliche und Erwachsene mit niedrigem Sozialstatus bzw. mit Migrationshintergrund sind weniger häufig im Sportverein zu finden, das betrifft das weibliche Geschlecht häufiger (Burrmann und Mutz 2017; Mutz 2020; Schmidt 2020; Wolff und Rütten 2013). Selbst bei niedrigschwelligen und kostengünstigen Angeboten zeigt sich eine soziale Ungleichheit. Im Kontext dieser Problematik haben die Sportbünde spezielle Förderprogramme wie „Integration durch Sport“, „mehr Migrantinnen im Sport“ und Einstiege für Menschen mit Fluchthintergrund kreiert (z. B. „Willkommen im Sport“) (Deutscher Olympischer Sportbund 2021).

Angesichts der Bedeutung der motorischen Entwicklung für die Entwicklung der Gesamtpersönlichkeit (Michel et al. 2018; Schwarz 2014), der Selbstverwirklichung und Teilhabe von Kindern und Jugendlichen (Bahr et al. 2012; Ortega et al. 2008), beschäftigt sich der vorliegende Artikel mit dem Potenzial des Sportvereins für die motorische Entwicklung. Konkret wird der Fragestellung nachgegangen, inwieweit es Unterschiede in der motorischen Entwicklung zwischen konstant aktiven Sportvereinsmitgliedern und dauerhaft Sportvereinsabstinenten gibt.

## Methodik

### Design der Motorik-Modul-Längsschnittstudie (MoMo)

Die MoMo-Studie ist ein Verbundvorhaben des Karlsruher Instituts für Technologie (KIT), der Pädagogischen Hochschule Karlsruhe (PH KA) und dem Robert Koch-Institut (RKI) in Berlin (Woll et al. 2017, 2021). Sie ist ein Vertiefungsmodul der repräsentativen KiGGS-Studie, einer Langzeitstudie zur gesundheitlichen Lage der Kinder und Jugendlichen in Deutschland (Kurth et al. 2008). Beide Studien sind analog zueinander mit einem quer- und einem längsschnittlichen Studienarm im Kohorten-Sequenzdesign angelegt. Ziel der MoMo-Studie ist es, bevölkerungsbezogene Daten zum Ist-Stand und zur Entwicklung der motorischen Leistungsfähigkeit und körperlich-sportlichen Aktivität von Kindern, Jugendlichen und jungen Erwachsenen in Deutschland zu liefern und ihre Einflussfaktoren zu untersuchen. Die MoMo-Teilnehmenden stellen eine randomisiert gezogene Unterstichprobe der KiGGS-Gesamtstichprobe des RKI dar, die in Kooperation mit dem GESIS – Leibniz-Institut für Sozialwissenschaften gezogen wurde (Woll et al. 2017). Drei Erhebungswellen wurden bereits abgeschlossen (MoMo T1: 2003–2006; MoMo T2: 2009–2012; MoMo T3: 2014–2017) und eine vierte dauert an. Der vorliegende Artikel beinhaltet Daten aus dem längsschnittlichen Studienarm aller abgeschlossenen Erhebungswellen (2003–2017).

### Teilnehmende

Die MoMo-Teilnehmenden wurden individuell kontaktiert und zu wohnortnah liegenden Testräumen eingeladen. Die Erhebungen fanden als Einzeltests statt, das heißt die Testteilnehmenden wurden über die gesamte Dauer von einem qualifizierten Testleitenden begleitet. Die Längsschnittstichprobe umfasst brutto insgesamt *N* = 4054 Teilnehmende. Von der Bruttostichprobe haben *N* = 1407 an allen drei Messzeitpunkten an der MoMo-Studie teilgenommen, es handelt sich also jeweils um dieselben Teilnehmenden. Von den *N* = 1407 Teilnehmenden haben *N* = 1092 Teilnehmende eine motorische Diagnostik zu allen drei Erhebungswellen absolviert. Aus diesen 1092 Teilnehmenden wurden all diejenigen mit konstanter Mitgliedschaft und (Nicht‑)Mitgliedschaft im Sportverein über drei Messwellen ausgewählt; dies entspricht 46 % der Gesamtstichprobe mit vorhandenen Daten zur motorischen Leistungsfähigkeit (*N* = 498).

Zum ersten Messzeitpunkt (T1) war die Stichprobe 8,9 ± 3,8 Jahre alt (Min: 4; Max: 17), zu T2 15,1 ± 3,9 (Min: 10; Max 24), zu T3 20,3 ± 4,0 (Min: 14; Max: 30). Die 498 Teilnehmer*innen (46 % männlich, 54 % weiblich) wurden kriteriengeleitet in drei verschiedene Gruppen aufgeteilt: über drei Messwellen konstante Sportvereinsmitglieder mit konstantem Wettkampfengagement (15 %), konstante Sportvereinsmitglieder ohne konstantes Wettkampfengagement (53 %) sowie konstante Sportvereinsabstinente (32 %). Sportvereinseinsteigende, Sportvereinsaussteigende und Sportvereinswiedereinsteigende (*n* = 594) wurden aufgrund von Unschärfen bezüglich der Dauer des Sportvereins-Engagement nicht einbezogen. Der besseren Lesbarkeit halber werden die drei Gruppen im Folgenden als „Sportvereinsmitglieder mit Wettkampf“, „Sportvereinsmitglieder ohne Wettkampf“ und „Sportvereinsabstinente“ bezeichnet (Tab. 1).

**Table 1:** 

	Sportvereinsmitglieder mit Wettkampf (*n* = 74)	Sportvereinsmitglieder ohne Wettkampf (*n* = 265)	Sportvereinsabstinente (*n* = 159)
*Männlich (%) | Weiblich (%)*	43 (58 %) | 31 (42 %)	131 (49 %) | 134 (51 %)	56 (35 %) | 103 (65 %)
*Sozioökonomischer Status (SES)*			
Niedrig (%)	1 (1 %)	8 (3 %)	30 (19 %)
Mittel (%)	54 (73 %)	165 (62 %)	98 (62 %)
Hoch (%)	19 (26 %)	92 (35 %)	31 (19 %)
*Körpermaße*			
Untergewicht (%)	4 (5 %)	19 (7 %)	15 (9 %)
Normalgewicht (%)	69 (93 %)	228 (87 %)	121 (76 %)
Übergewicht (%)	1 (1 %)	16 (6 %)	23 (15 %)
*Sportminuten im Sportverein/Woche*	186 ± 134	126 ± 85	0 ± 0
*Sportminuten im Freizeitsport/Woche*	75 ± 94	68 ± 104	79 ± 131

Die Daten sind Anzahl und Prozent (%, Abweichung zu 100 % ergibt sich aus der Rundung) oder Mittelwerte (MW) ± Standardabweichung (SD); Sozialstatus (SES) nach Lampert et al. (2018)

Betrachtet man das Spektrum der Sportarten, so ist die Vielfalt im organisierten Sport mit zwischen 43 und 66 verschiedenen Sportarten etwas größer als im Freizeitsport mit 40 bis 57 genannten Sportarten. Sportvereinsmitglieder betreiben in einem ähnlichen wöchentlichen Umfang wie Sportvereinsabstinente zusätzlich nicht-organisierten Freizeitsport. Die in diese Untersuchung einbezogenen Sportvereinsmitglieder berichteten folgende Sportarten am häufigsten (Tab. 2).

**Table 2:** 

Sportverein häufigste Sportarten	MoMo T1 (2003–2006)	MoMo T2 (2009–2012)	MoMo T3 (2014–2017)
Männlich	Fußball, Schwimmen und Leichtathletik	Fußball, Schwimmen und Leichtathletik	Fußball, Volleyball und Handball
Weiblich	Turnen, Tanzen und Schwimmen	Tanzen, Schwimmen und Leichtathletik	Tanzen, Reiten und Fußball

### Testaufgaben zur motorischen Leistungsfähigkeit

Die Erfassung der motorischen Leistungsfähigkeit erfolgte fähigkeitsorientiert. Im Rahmen des mehrdimensionalen MoMo-Testprofils wurden sechs Testaufgaben einbezogen, die eine Gesamtaussage zu Kondition sowie zur Koordination zulassen (Worth et al. 2021). Die konditionellen Fähigkeiten wurden anhand von Standweitsprung, Liegestütz und Fahrrad-Ausdauertest (PWC 170) ermittelt, die koordinativen Fähigkeiten anhand des seitlichen Hin- und Herspringens, des Einbeinstands und des Balancierens rückwärts (Worth et al. 2021). Aufgrund des dominanten Einflusses von Geschlecht und Alter auf die Entwicklung der motorischen Fähigkeiten über die Zeit, erfolgte die gruppenvergleichende Berechnung anhand von alters- und geschlechtsadjustierten Perzentilwerten. Die Datengrundlage dieser Perzentile lieferten *N* = 3742 Teilnehmende der repräsentativen MoMo-Gesamtstichprobe der Welle 1, die Berechnung erfolgte mit der LMS-Transformationsmethode von Cole und Green (Details der Berechnung nachzulesen in: Niessner et al. 2020). Die Werte variieren zwischen einem Perzentilwert von 1 (schlechtester Wert) und 99 (bester Wert).

### Erfassung der körperlich-sportlichen Aktivität

Die habituelle körperlich-sportliche Aktivität wurde fragebogenbasiert durch den MoMo-Aktivitätsfragebogen (MoMo-AFB) erhoben (Bös et al. 2009). Innerhalb verschiedener Domänen (Schule, Sportverein, Freizeit, Alltag) wurden die Sportart, der Umfang und die Intensität abgefragt. Die Erfassung der körperlich-sportlichen Aktivität im Sportverein erfolgte zunächst anhand der Einstiegsfrage zur Vereinsmitgliedschaft: Bist du Mitglied in einem Sportverein? Es folgten die Antwortmöglichkeiten: a) Nein, ich war noch nie Mitglied in einem Sportverein, b) Nein, ich bin derzeit kein Mitglied aber früher schon, c/d) Ja, ich bin derzeit Mitglied in einem Sportverein/mehreren Sportvereinen. Beim Ausfüllen der Sportarten im Sportverein wurde die Wettkampfteilnahme bei der Erfassung der Sportarten per Checkbox mit erhoben. Der Fragebogen besitzt eine akzeptable Reliabilität von 0,68 (Jekauc et al. 2013).

### Erfassung sozioökonomischer Status

Der sozioökonomische Status (SES) wurde anhand eines Index erfasst, der den Bildungsstand und die berufliche Stellung der Eltern sowie die Einkommenssituation (Nettoäquivalenzeinkommen des Haushalts) einbezieht. Für jeden Bereich werden Werte von eins bis sieben vergeben, die aufsummiert einen Gesamtindex bilden, der zwischen den Werten drei und 21 variieren kann. Bei der Analyse wurde eine Einteilung des Gesamtindex in fünf Quintile vorgenommen: Das erste Quintil bildet die niedrige Statusgruppe, das zweite bis vierte Quintil die mittlere und das fünfte Quintil die hohe Statusgruppe (Lampert et al. 2018). Für die deskriptiven Darstellungen werden die Statusgruppen verwendet.

### Vorgehensweise und statistische Analyse

Sämtliche statistische Auswertungen erfolgten mit SPSS, 26. Im Rahmen der Auswertung werden Mittelwerte in Verbindung mit Standardabweichungen bzw. 95 %-Konfidenzintervallen (95 % KI) für alle Erhebungswellen dargestellt. Ein Unterschied zwischen den Gruppen zu einem Messzeitpunkt ist dann erkennbar, wenn sich die dargestellten Konfidenzintervalle nicht überlappen. Des Weiteren wurde eine Varianzanalyse mit Messwiederholung eingesetzt, um Unterschiede in der Entwicklung über die drei Messzeitpunkte hinweg zu erkennen. Für die Darstellung der Größe des Effekts wird der Eta-Koeffizient angegeben und zusätzlich in f umgerechnet, um eine Einordnung vornehmen zu können. Dabei steht 0,1 für einen kleinen Effekt, 0,25 für einen mittleren und 0,4 für einen großen Effekt (Cohen 1988). Die alters- und geschlechtsadjustierten Perzentile der Kondition und Koordination wurden in der Varianzanalyse mit dem Gesamtscore des Sozialstatus (SES) als Kovariate betrachtet, weil dieser sich innerhalb der Gruppen unterschied. Das Signifikanzniveau wurde auf α = 0,05 festgelegt.

## Ergebnisse

### Merkmale der Stichprobe

In der Gruppe der 159 Sportvereinsabstinenten sind weibliche Studienteilnehmende mit 65 % überrepräsentiert (siehe Tab. 1). Das Geschlechterverhältnis bei Sportvereinsmitgliedern ohne Wettkampfengagement ist demgegenüber nahezu ausgeglichen, während es sich in der Gruppe der Sportvereinsmitglieder mit Wettkampfengagement dreht: hier überwiegen männliche Studienteilnehmende mit 58 %. In Bezug auf den Sozialstatus sind die Teilnehmenden mit niedrigem Sozialstatus in beiden dauerhaft im Sportverein organisierten Gruppen deutlich unterrepräsentiert mit 1 und 3 %. Die zum ersten Messzeitpunkt berichtete körperlich-sportliche Aktivität im Freizeitsport zeigt, dass sich alle drei beobachteten Gruppen in ähnlicher Weise auch in der Freizeit bewegen.

### Motorische Leistungsfähigkeit

Insgesamt betrachtet, ist die Entwicklung der koordinativen und konditionellen Fähigkeiten bei Sportvereinsmitgliedern als signifikant besser zu beurteilen im Vergleich zu Sportvereinsabstinenten (Modell Koordination * Sportverein: df = 3,870 | F = 2,931 | *p* = 0,021 | ETA = 0,015 | f = 0,123; Modell Kondition * Sportverein: df = 4 | F = 3,794 | *p* = 0,005 | ETA = 0,048 | f = 0,225) (siehe Tab. 3).

**Table 3:** 

	MoMo T1 (2003–2006)	MoMo T2 (2009–2012)	MoMo T3 (2014–2017)
*Koordination MW [95* *% KI]*			
Sportvereinsabstinente	34 [30–38]	52 [48–57]	51 [47–55]
Sportvereinsmitglieder ohne Wettkampf	42 [39–45]	68 [65–71]	66 [63–69]
Sportvereinsmitglieder mit Wettkampf	49 [43–54]	68 [62–73]	71 [65–76]
Modell Koordination^a^	df = 1,935 | F = 7,784 | *p* = 0,001 | ETA = 0,020 | f = 0,500		
Modell Koordination * SES^a^	df = 1,935 | F = 3,340 | *p* = 0,038 | ETA = 0,009 | f = 0,095		
Modell Koordination * Sportverein^a^	df = 3,870 | F = 2,931 | *p* = 0,021 | ETA = 0,015 | f = 0,123		
*Kondition MW [95* *% KI]*			
Sportvereinsabstinente	48 [43–53]	43 [37–49]	44 [39–50]
Sportvereinsmitglieder ohne Wettkampf	50 [46–54]	57 [52–61]	57 [53–61]
Sportvereinsmitglieder mit Wettkampf	59 [52–65]	66 [59–73]	63 [56–69]
Modell Kondition^b^	df = 2 | F = 0,854 | *p* = 0,427 | ETA = 0,006 | f = 0,078		
Modell Kondition * SES^b^	df = 2| F = 0,329 | *p* = 0,726 | ETA = 0,002 | f = 0,045		
Modell Kondition * Sportverein^b^	df = 4 | F = 3,794 | *p* = 0,005 | ETA = 0,048 | f = 0,225		

Die Daten sind geschätzte mittlere Randmittel der alters- und geschlechtsadjustierten Perzentile des Modells, 95 %-Konfidenzintervalle (KI)

^a^Keine Sphärizität gegeben, daher wurden die Freiheitsgrade mittels Greenhouse-Geisser angepasst

^b^Sphärizität gegeben, keine Anpassung der Freiheitsgrade notwendig

### Ergebnisse Koordination

**Figure 2:**
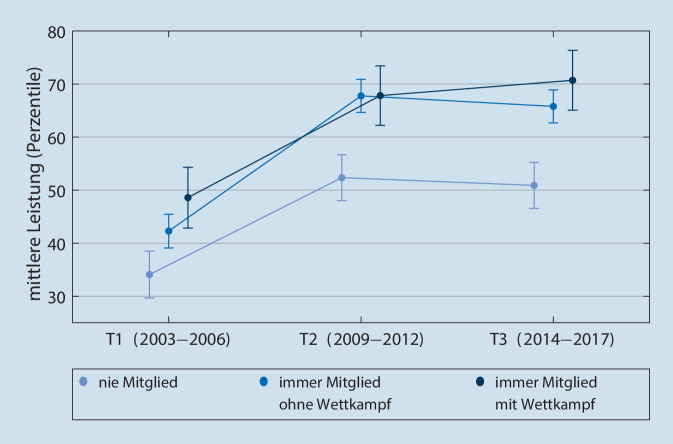

Herangezogen werden die gemittelten alters- und geschlechtsadjustierten Perzentile zu den Ergebnissen der Testaufgaben Einbeinstand, Balancieren rückwärts sowie Seitliches Hin- und Herspringen (Abb. 2; Tab. 3).

Insgesamt ist die Entwicklung der koordinativen Fähigkeiten der Sportvereinsmitglieder signifikant besser als die der Vereinsabstinenten (Eta-Koeffizient = 0,015, f = 0,12, das entspricht einem geringen Effekt). Das Ausgangsniveau der Sportvereinsabstinenten lag signifikant unterhalb der Sportvereinsmitglieder ohne Wettkampf und der Sportvereinsmitglieder mit Wettkampf. Von T1 (2003–2006) zu T2 (2009–2012) entwickeln sich alle drei Gruppen signifikant positiv. Von T2 (2009–2012) bis T3 (2014–2017) stagniert die Entwicklung in den drei Gruppen überwiegend.

Um dies exemplarisch anhand von Rohwerten in Bezug auf das Seitliche Hin- und Herspringen zu verdeutlichen: Zu T3 springen die männlichen Sportvereinsmitglieder mit Wettkampfengagement im Durchschnitt knapp zwei Sprünge mehr als Sportvereinsmitglieder ohne Wettkampfengagement und sieben Sprünge mehr als Sportvereinsabstinente. Analog dazu springen die weiblichen Sportvereinsmitglieder mit Wettkampfengagement knapp vier Sprünge mehr als Sportvereinsmitglieder ohne Wettkampfengagement und acht Sprünge mehr als Vereinsabstinente.

### Ergebnisse Kondition

**Figure 3:**
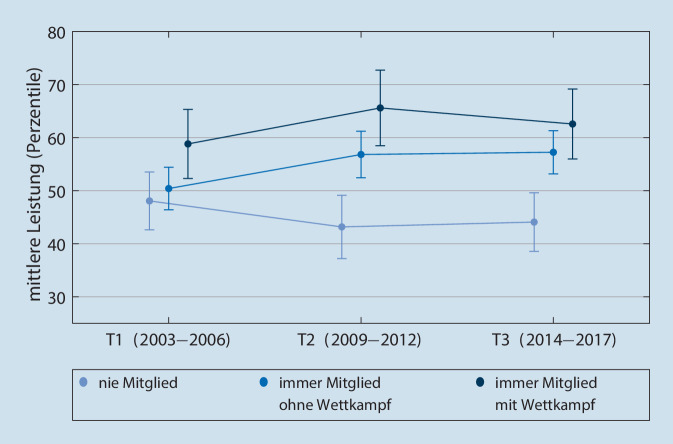

Hierfür werden die gemittelten alters- und geschlechtsadjustierten Perzentile zu den Ergebnissen der Testaufgaben Standweitsprung, Liegestütze und Fahrrad-Ausdauertest (PWC 170) herangezogen (Abb. 3).

Insgesamt ist die Entwicklung der konditionellen Fähigkeiten für dauerhaft im Sportverein aktive Sportler*innen signifikant besser als die der Vereinsabstinenten (Eta-Koeffizient = 0,048, f = 0,22, das entspricht einem mittleren Effekt). Das Ausgangsniveau der drei beobachteten Gruppen unterscheidet sich nicht signifikant. Von T1 zu T2 verbessern sich beide im Sportverein aktiven Gruppen hinsichtlich ihrer konditionellen Fähigkeiten und die Gruppe der Vereinsabstinenten verschlechtert sich parallel. Beide Gruppen Sportvereinsmitglieder sind konditionell signifikant besser zu T2 als die Sportvereinsabstinenten und Sportvereinsmitglieder mit Wettkampf. Von T2 zu T3 stagniert die Entwicklung, sodass die beiden im Sportverein aktiven Gruppen signifikant besser bleiben im Vergleich zu den Vereinsabstinenten.

Dies bedeutet exemplarisch anhand von Rohwerten in Bezug auf den Standweitsprung erläutert: Zu T3 springen die männlichen Sportvereinsmitglieder mit Wettkampfengagement 6 cm weiter als Sportvereinsmitglieder ohne Wettkampfengagement und 16 cm weiter als Vereinsabstinente. Analog dazu springen die weiblichen Sportvereinsmitglieder mit Wettkampfengagement 12 cm weiter als Sportvereinsmitglieder ohne Wettkampfengagement und 30 cm weiter als Vereinsabstinente.

## Diskussion

Im Rahmen der vorliegenden Studie konnte gezeigt werden, dass dauerhaft im Sportverein engagierte Teilnehmende der MoMo-Studie über eine bessere motorische Leistungsentwicklung in den Bereichen Koordination und Kondition verfügen als dauerhaft nicht im Sportverein Aktive. Das Wettkampfengagement ist in dieser Studie mit keinem relevanten zusätzlichen Vorteil für die motorische Entwicklung verbunden, auch wenn die Entwicklung im Bereich Koordination und Kondition auf etwas höherem Niveau stattfindet, aber sie unterscheidet sich innerhalb der beiden Gruppen Sportvereinsmitglieder mit und ohne Wettkampfengagement nicht signifikant.

Nicht im Sportverein sportlich aktiv zu sein ist in der untersuchten Stichprobe nicht mit sportlich inaktiv gleichzusetzen. Die Settings Sportverein und Freizeitsport scheinen sich nicht gegenseitig zu verdrängen (siehe Tab. 1): Alle drei Gruppen treiben in der Freizeit vergleichbar viel Sport, dies zeigt sich auch für T2 und T3. Die hohen Standardabweichungen weisen jedoch auf die begrenzte Aussagekraft des Mittelwerts zum Freizeitsport hin (siehe Tab. 1).

Betrachtet man die Entwicklung über die Zeit, so scheint das Potenzial des Sportvereins für die motorische Entwicklung in Kindheit und Jugend ausgeprägter zu sein als im (jungen) Erwachsenenalter (siehe Abb. 1 und 2). Dies kann darin begründet sein, dass sich die motorischen Fähigkeiten im Kindes- und Jugendalter mit geringerem Aufwand steigern lassen als in anderen Lebensphasen: Die für Kindheit und Jugend „normale“ Entwicklung ist bei den meisten motorischen Fähigkeiten eine kontinuierliche Steigerung (Albrecht 2016; Bös 1994), bedingt durch Wachstum, Reife sowie Lern- und Trainingsprozesse. Der Höhepunkt der motorischen Leistungsfähigkeit ist um das 18. Lebensjahr zu erwarten, gefolgt von einer Stagnation und einem Leistungsrückgang im Erwachsenenalter (Albrecht 2016; Bös 1994). Bemerkenswert sind im koordinativen Bereich die unterschiedlichen Ausgangsniveaus, welche bereits in jungen Jahren bestehen. Ob dieser Unterschied durch vorangegangenes Engagement im Sportverein miterklärt werden kann oder ob andere Faktoren hierfür verantwortlich sind, lässt sich anhand der vorliegenden Studie nicht beantworten.

Studien zeigen, dass Kinder und Jugendliche, die Sport im Sportverein treiben, dabei höhere Intensitäten erzielen als während nicht-organisierter körperlich-sportlicher Aktivität (Bös et al. 2009; Will et al. 2016). Internationale Studien erklären die gesundheitsprotektive Wirkung der regelmäßigen Teilnahme am organisierten Sport auch mit dem häufigeren Erreichen der Aktivitätsempfehlungen sowie einer positiven Beeinflussung weiterer Präventionsfelder wie der Ernährung und des Medienkonsums (Kokko et al. 2019; Vella et al. 2013). Mehr trainingswirksame körperlich-sportliche Aktivität kann im Rahmen der vorliegenden Studie die bessere motorische Entwicklung bei den dauerhaften Sportvereinsmitgliedern mit erklären.

### Geschlecht und Sozialstatus

Die vorliegende Studie bestätigt denselben geschlechtsbezogenen Trend, der sich auch in vielen anderen Studien zeigt: Mädchen und junge Frauen bleiben dem Sportverein häufiger fern (Breuer et al. 2015). Der eingangs beschriebene soziale Gradient in Bezug auf die Partizipation im Sportverein bestätigt sich ebenfalls und ist mit 1 und 3 % Teilnehmenden mit niedrigem Sozialstatus in den im Sportverein aktiven Gruppen in dieser längsschnittlichen Studie auffälliger als in den meisten Querschnittstudien. Trotz der beschriebenen Verfügbarkeit und der vergleichsweise geringen Kosten u. a. aufgrund der Möglichkeit staatlicher Co-Finanzierung durch Teilhabeleistungen engagieren sich junge Menschen mit niedrigem Sozialstatus sehr selten kontinuierlich in Sportvereinen. Hier stellt sich die Frage, ob es weitere Zugangsbarrieren gibt, das Angebot als offen und attraktiv wahrgenommen wird. Aus Public Health-Sicht wäre es wünschenswert, vermehrt Mädchen und Frauen und Teilnehmende mit niedrigem Sozialstatus für das gemeinsame Sporttreiben im Sportverein bzw. für organisierte Angebote zu gewinnen (Vella et al. 2013). Es liegen bereits Erkenntnisse vor, wie das innerhalb der Sportvereine gelingen kann (Wolff und Rütten 2013). Ein Ansatz ist die Intensivierung der geförderten Kooperationen Schule-Sportverein. Die Studienlage zeigt, dass bei schulnahen Maßnahmen zur Bewegungsförderung, wie beispielsweise außercurriculare Sport-AGs, Mädchen aus Familien mit niedrigem Sozialstatus oder mit Migrationshintergrund häufiger partizipieren als an reinen Sportvereinsangeboten (Schmidt et al. 2016; Mutz 2020).

### Unterstützungsstrategien zur Förderung der Partizipation

Durch finanziell zumeist verträgliche Mitgliedsbeiträge haben Sportvereine eher auf den ersten Blick niedrige Zugangshürden. Zusätzlich kann die Mitgliedschaft für Kinder und Jugendliche aus einkommensschwachen Familien staatlich bezuschusst werden im Rahmen von Teilhabeleistungen. Es wäre wichtig zu wissen, ob das von den Familien mit entsprechendem Unterstützungsbedarf in Anspruch genommen wird oder ob es eine Verwaltungshürde darstellt. Hier sind die Kinder auf das Engagement ihrer Eltern angewiesen. Die vorliegende Studie zeigt, dass die (dauerhafte) Teilhabe von Kindern und Jugendlichen aus Familien mit niedrigem Sozialstatus in Sportvereinen niedrig ist. Schwer erreichbare Zielgruppen (niedriger Sozialstatus, zugewandert) über längere Zeit zu binden, ist als Herausforderung zu sehen (Burrmann und Mutz 2017). Es existieren Brückenangebote in die Sportvereine: beispielsweise die „Kooperation Schule – Sportverein“ oder Angebote in Ganztagsschulen und Sport-AGs, die von Vereinsseite durchgeführt werden (Burrmann und Mutz 2017; Mutz 2020; Segel 2014). Vor dem Hintergrund der vorliegenden Daten erscheint es sinnvoll, diese Brückenangebote auszuweiten.

Sportvereine, die sich auf den Weg machen wollen, vermehrt schwer erreichbare Zielgruppen als sportlich aktive Mitglieder zu gewinnen, sollten die Stärken und Ressourcen dieser Zielgruppen einbeziehen (Empowerment) und Angebote unter ihrer Beteiligung entstehen lassen (Wright et al. 2010). Studien zeigen, dass hierfür Unterstützung von außen notwendig sein kann (Wolff und Rütten 2013). Es ist weiterhin zu erproben, welche Ansätze sich für das jeweilige Geschlecht und die verschiedenen Altersgruppen eignen. Die Gewinnung und Qualifizierung der unterrepräsentierten Gruppen als Übungsleiter*innen kann beispielweise zu Multiplikatoreffekten führen (Wolff und Rütten 2013). Ein partizipativer Peer-to-peer-Ansatz ist ebenfalls denkbar: Im Sportverein aktive Mädchen und junge Frauen mit Zuwanderungshintergrund informieren über Struktur und Angebote des Sportvereins (auch in der jeweiligen Muttersprache) und fungieren als „role models“.

### Unterstützungsstrategien aufgrund der Covid-19-Pandemie

Auch wenn die einbezogenen Daten vor der Pandemie erhoben wurden, werden die Ergebnisse im Folgenden im Kontext der Covid-19-Pandemie reflektiert. Die pandemiebedingte Schließung der Sportstätten und Sportvereine wurde von Kindern, Jugendlichen und Erwachsenen als sehr einschränkend wahrgenommen (Deutsches Kinderhilfswerk 2021). Es zeigte sich, dass das Wegfallen des organisierten Sports im Laufe der Pandemie zunehmend mit einer bevölkerungsbezogen erheblichen Einbuße an Bewegungszeit verbunden war (Schmidt et al. 2021). Inwieweit es dadurch zu Einbußen bei der motorischen Entwicklung gekommen ist, muss sich in zukünftigen Studien zeigen. Diejenigen in besserer Wohnumgebung und mit Zugang zu Grünflächen oder einem eigenen Garten konnten das besser kompensieren (Schmidt et al. 2020). Im zweiten Lockdown im Winter 2020/2021 fiel die körperliche Aktivität jedoch bevölkerungsbezogen auf ein sehr niedriges Niveau (Schmidt et al. 2021). Die bereits vorhandene Ungleichheit an Zugängen zu Bewegung und Sport wurde durch die Pandemie verstärkt (Schmidt et al. 2020). Hier setzt die Überlegung an, dass Sportvereine im Rahmen ihrer Möglichkeiten mit ihren Angeboten dabei unterstützen, dass sich die vorhandene Ungleichheit nicht weiter verstärkt. Wenn nach der Corona-Pandemie jedes Kind ein Jahr lang kostenlos bei einem Sportverein mitmachen könnte, würden 61 % der Kinder und Jugendlichen dieses Angebot auf jeden Fall wahrnehmen, weitere 25 % würden dies wahrscheinlich tun (Deutsches Kinderhilfswerk 2021).

### Schwächen und Stärken der Studie

Die reduzierte Gruppenbildung kann methodisch kritisch diskutiert werden gegenüber einer Darstellung mit allen „Zwischengruppierungen“ an Sportvereinsaussteiger*innen und -(wieder)einsteiger*innen. Das Vorgehen ist mit einem Verlust an statistischer Aussagekraft verbunden. Dies wurde im vorliegenden Fall einer weiter differenzierenden Darstellung vorgezogen, da die Zeitdauer des Sportvereinsengagements bei allen „Zwischengruppierungen“ aufgrund der weit auseinanderliegenden Messzeitpunkte zu unpräzise ist. Die Analyse der betriebenen Sportarten gibt lediglich einen groben Überblick und quantitative Ausprägungen pro Sportart wurden dabei nicht berücksichtigt. Kausale Zusammenhänge, welche die Wirkungsmechanismen zwischen der Sportvereinsaktivität und der motorischen Leistungsfähigkeit aufdecken, können mit den durchgeführten Verfahren nicht analysiert werden.

Eine Stärke der vorliegenden Studie ist die auf Deutschland bezogene Repräsentativität der MoMo-Stichprobe. Die Anwerbung der Teilnehmenden erfolgte mit dem Ziel, eine bezüglich Alter, Geschlecht, Region, Migrationshintergrund und Bildungsstatus ausgewogene Stichprobe zu erhalten. Die Auswahl von validierten und reliablen Tests und Fragebogens sowie die Durchführung der Untersuchung mit qualifizierten Testleiter*innen sind als weitere Stärken der Studie hervorzuheben.

### Fazit

Es ist eine gesamtgesellschaftliche Aufgabe, Kindern und Jugendlichen geeignete Rahmenbedingungen für ein gesundes Aufwachsen zu ermöglichen. Neben regelmäßiger körperlich-sportlicher Aktivität in der Freizeit bestätigt die vorliegende Auswertung die Annahme, dass langfristig betriebene organisierte Sportvereinsaktivität mit einer positiven motorischen Entwicklung einhergeht und somit auch wichtige Impulse für die Entwicklung der Gesamtpersönlichkeit beiträgt (Krug et al. 2019; Ortega et al. 2008). Insgesamt untermauern die Ergebnisse die Wichtigkeit der Sportvereine für die motorische Entwicklung von Kindern, Jugendlichen und jungen Erwachsenen in Deutschland. Die vermehrte und langfristige Teilhabe von Kindern, Jugendlichen und jungen Erwachsenen aus Familien mit niedrigem Sozialstatus am organisierten Sport stellt ein wichtiges Ziel auch im Sinne der Gesundheitsförderung dar.
